# VLDL from Metabolic Syndrome Individuals Enhanced Lipid Accumulation in Atria with Association of Susceptibility to Atrial Fibrillation

**DOI:** 10.3390/ijms17010134

**Published:** 2016-01-20

**Authors:** Hsiang-Chun Lee, Hsin-Ting Lin, Liang-Yin Ke, Chi Wei, Yi-Lin Hsiao, Chih-Sheng Chu, Wen-Ter Lai, Shyi-Jang Shin, Chu-Huang Chen, Sheng-Hsiung Sheu, Bin-Nan Wu

**Affiliations:** 1 Division of Cardiology, Department of Internal Medicine, Kaohsiung Medical University Hospital, Kaohsiung 807, Taiwan; hclee@kmu.edu.tw (H.-C.L.); hsintinglin2007@gmail.com (H.-T.L.); r00b22042@ntu.edu.tw (C.W.); irpu10.yls@gmail.com (Y.-L.H.); jujuson993@gmail.com (C.-S.C.); wtlai@kmu.edu.tw (W.-T.L.); 2 Graduate Institute of Medicine, College of Medicine, Kaohsiung Medical University, Kaohsiung 807, Taiwan; 3 Department of Internal Medicine, Faculty of Medicine, College of Medicine, Kaohsiung Medical University, Kaohsiung 807, Taiwan; sjshin@kmu.edu.tw; 4 Center for Lipid Biosciences, Kaohsiung Medical University Hospital, Kaohsiung 807, Taiwan; kly@cc.kmu.edu.tw (L.-Y.K.); cchen@texasheart.org (C.-H.C.); 5 Lipid Science and Aging Research Center, Kaohsiung Medical University, Kaohsiung 807, Taiwan; 6 Vascular and Medicinal Research, Texas Heart Institute, Houston, TX 77030, USA; 7 New York Heart Research Foundation, Mineola, NY 11501, USA; 8 Lipid and Glycoimmune Research Center, Changhua Christian Hospital, Changhua 500, Taiwan; 9 Department of Pharmacology, College of Medicine, Kaohsiung Medical University, Kaohsiung 807, Taiwan

**Keywords:** atrial fibrillation, lipotoxicity, metabolic syndrome, very-low-density lipoprotein (VLDL)

## Abstract

Metabolic syndrome (MetS) represents a cluster of metabolic derangements. Dyslipidemia is an important factor in MetS and is related to atrial fibrillation (AF). We hypothesized that very low density lipoproteins (VLDL) in MetS (MetS-VLDL) may induce atrial dilatation and vulnerability to AF. VLDL was therefore separated from normal (normal-VLDL) and MetS individuals. Wild type C57BL/6 male mice were divided into control, normal-VLDL (nVLDL), and MetS-VLDL (msVLDL) groups. VLDL (15 µg/g) and equivalent volumes of saline were injected via tail vein three times a week for six consecutive weeks. Cardiac chamber size and function were measured by echocardiography. MetS-VLDL significantly caused left atrial dilation (control, *n* = 10, 1.64 ± 0.23 mm; nVLDL, *n* = 7, 1.84 ± 0.13 mm; msVLDL, *n* = 10, 2.18 ± 0.24 mm; *p* < 0.0001) at week 6, associated with decreased ejection fraction (control, *n* = 10, 62.5% ± 7.7%, *vs.* msVLDL, *n* = 10, 52.9% ± 9.6%; *p* < 0.05). Isoproterenol-challenge experiment resulted in AF in young msVLDL mice. Unprovoked AF occurred only in elderly msVLDL mice. Immunohistochemistry showed excess lipid accumulation and apoptosis in msVLDL mice atria. These findings suggest a pivotal role of VLDL in AF pathogenesis for MetS individuals.

## 1. Introduction

The incidence of atrial fibrillation (AF), the most common arrhythmia, is rising as the population ages [1]. Metabolic syndrome (MetS) represents a cluster of cardiovascular and metabolic derangements that include increased blood pressure, abdominal obesity, insulin resistance, elevated triglycerides (TG) and low high-density lipoprotein cholesterol (HDL-C). MetS has become a prevalent health problem in developed countries. Recent clinical studies suggest that MetS increases the risk and complication of AF and also the risk of recurrence after catheter ablation [2,3,4,5]. The exact mechanisms remain unclear. Effective prevention of AF is an unmet clinical need for MetS. The critical pathogenic factor of AF development in MetS remains undetermined.

A relationship between dyslipidemia and AF has emerged in recent years [6,7]. An 18.7 median year follow-up study in the United States associated higher total cholesterol and low-density lipoprotein cholesterol (LDL-C) with a lower incidence of AF but no independent association for HDL-C and TG [6]. Another clinical study with a median follow-up of 4.5 years in Japan associated low HDL-C with increased risk of new-onset AF in women but not men [7]. Although the association of dyslipidemia and AF is still debated, data on the beneficial role of statins or lipid-lowering therapy in AF are growing [8,9,10,11,12,13]. Of note, lipoprotein properties are reported to be severely impaired in male AF patients with greater oxidation and inflammation [14]. However, it is still unclear if lipoproteins directly contribute to AF pathogenesis.

Patients with MetS or type 2 diabetes often have increased plasma levels of TG and TG-derived very low density lipoproteins (VLDL) [15,16]. We previously showed that a negatively charged subfraction of VLDL in MetS patients was most toxic to human vascular endothelial cells and that the negatively charged VLDL was richer in MetS than healthy controls [17]. Moreover, VLDL from patients with AF is more highly glycated and more oxidized [18]. Therefore, we hypothesize that VLDL from MetS and healthy individuals exert different effects on atrial remodeling that may result in AF susceptibility.

Our current study confirmed that VLDL from MetS individuals (MetS-VLDL) was more cytotoxic to atrial myocytes (HL-1 cells) than VLDL from normal subjects. To examine whether cytotoxic MetS-VLDL could cause any atrial remodeling or even AF, we intravenously injected VLDL from healthy (normal-VLDL) or MetS individuals to mice. This model allowed us to examine the *in vivo* effects of VLDL on the atrium.

## 2. Results

### 2.1. Very Low Density Lipoproteins (VLDL) in MetS (MetS-VLDL) Increased Oxidative Stress and Cytotoxicity to HL-1 Atrial Myocytes

**Figure 1:**
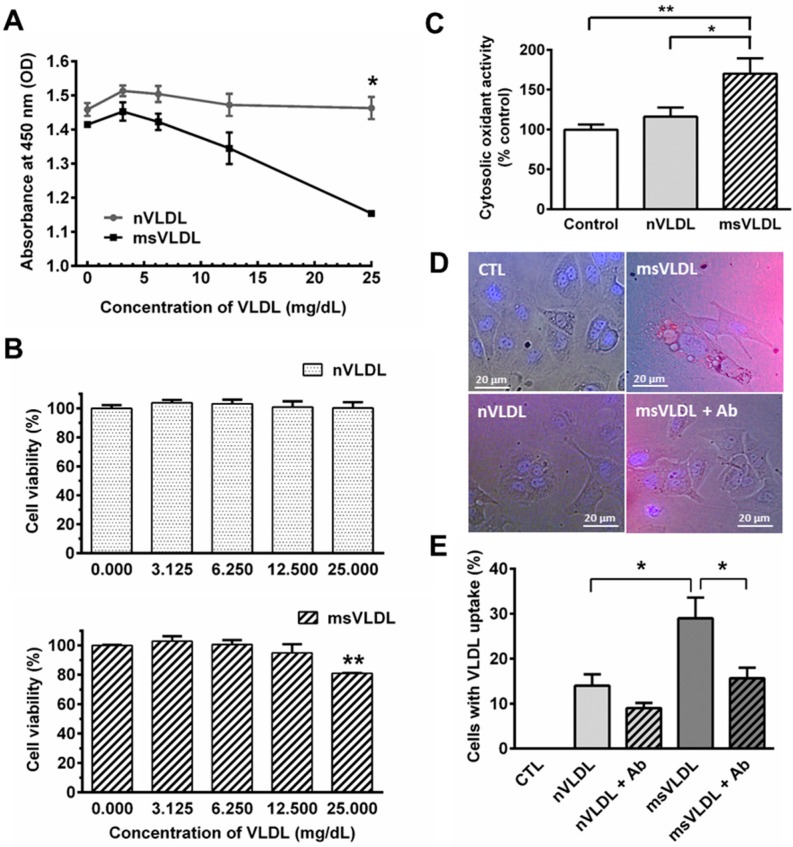
Metabolic syndrome-very low density lipoproteins (MetS-VLDL) is cytotoxic and provokes oxidative stress, with greater internalization in HL-1 atrial myocytes. (**A**) HL-1 cells were treated with normal-VLDL or MetS-VLDL at different test concentrations (3.125, 6.25, 12.5 and 25 mg/dL) for 24 h. OD values at 450 nm indicating viability were significantly lower in the msVLDL group (* *p* < 0.05; *n* = 4 for each group); (**B**) HL-1 cells treated with MetS-VLDL showed significantly reduced cell viability (% of control) with a concentration of 25 mg/mL (** *p* < 0.01; *n* = 4); (**C**) DCF fluorescence (excitation at 480 nm and emission at 520 nm) indicated total cytosolic oxidant activity (values of % control; *n* = 3 for each group). MetS-VLDL significantly increased oxidative stress at 25 mg/dL (* *p* < 0.05; ** *p* < 0.01); (**D**) Representative images for control, nVLDL, msVLDL, and msVLDL with VLDL receptor (VLDLR) antibody (msVLDL + Ab) groups (*n* = 3 for each group); (**E**) Internalization of DiI-labeled VLDL particles (red) increased in size and number in MetS-VLDL treated HL-1 cells (msVLDL) compared to normal-VLDL treated cells (nVLDL) (* *p* < 0.05, *n* = 3). Pre-treatment with VLDLR Ab for 24 h reduced internalization (* *p* < 0.05).

To identify the cytotoxic effect of VLDLs on cardiomyocytes, we exposed cultured HL-1 cells to different concentrations of normal-VLDL and MetS-VLDL, using a CCK-8 assay to determine cell viability. Optical density (OD) values decreased at 450 nm indicating reduction of cell viability. Normal-VLDL did not affect HL-1 cell viability at concentrations from 3.125 to 25 mg/dL after 24 h. MetS-VLDL, however, significantly reduced cell viability at concentrations of 25 mg/dL after 24 h (*p* < 0.01; Figure 1A,B). Reactive oxygen species (ROS) activity was significantly increased by 25 mg/dL MetS-VLDL incubation compared with control and normal-VLDL (*n* = 4, *p* < 0.01 and *p* < 0.05, respectively; Figure 1C), suggesting that only MetS-VLDL caused oxidative stress to HL-1 cells.

### 2.2. Uptake of VLDLs by VLDL Receptors of Atrial Myocytes

We labeled VLDL particles with DiI to investigate the internalization of normal-VLDL and MetS-VLDL in HL-1 atrial myocytes (Figure 1D). To determine VLDLs internalization through the VLDL receptor (VLDLR), we designed five test groups including control, nVLDL, nVLDL with pretreatment of VLDLR antibody, msVLDL, and msVLDL with pretreatment of VLDLR antibody. After 24 h incubation, the internalization of DiI-labeled VLDL particles (red) was significantly different among groups (*p* = 0.0071). The size and number were increased in msVLDL HL-1 cells compared to nVLDL cells (*n* = 3, *p* < 0.05). Pre-treatment by VLDLR antibody for 24 h significantly reduced the internalization of msVLDL, but not of nVLDL (*n* = 3, *p* < 0.05; Figure 1E).

### 2.3. Both Normal-VLDL and MetS-VLDL Increased Left Ventricular (LV) Mass but Only MetS-VLDL Caused Left Atrial Dilation

**Figure 2:**
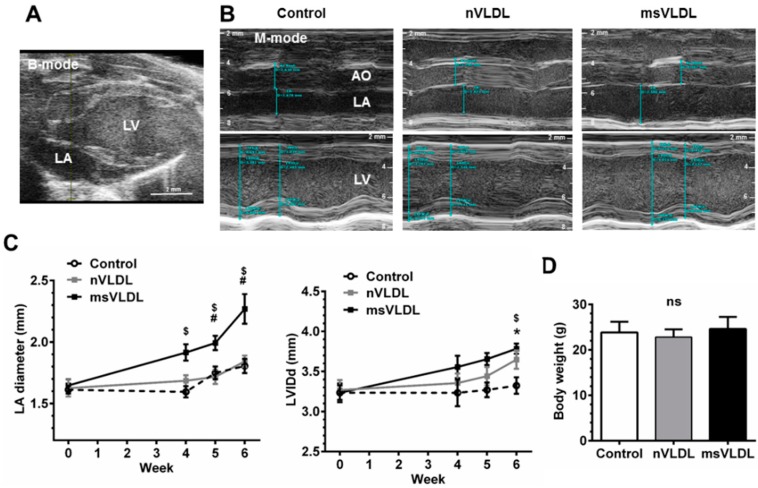
Both VLDLs caused LV dilation but only MetS-VLDL caused left atrial dilation. (**A**) Echocardiography of murine heart. Left atrium (LA) and left ventricle (LV) were identified in B-mode; (**B**) M-mode images for measurements of diameters of aortic root (AO), LA and LV. LA was significantly enlarged in the MetS-VLDL injection group (msVLDL) (*n* = 6) but not in the normal-VLDL injection group (nVLDL) (*n* = 7) or the control group (*n* = 5); (**C**) Significant LA enlargement developed as early as 4–6 weeks after injection in the msVLDL group. LV dilatation developed significantly until 6 weeks. (msVLDL *vs.* control, $ *p* < 0.05; msVLDL *vs.* nVLDL, # *p* < 0.05; nVLDL *vs.* control, * *p* < 0.05); (**D**) No significant difference in body weight of the groups.

We further hypothesized that cytotoxic MetS-VLDL can cause atrial remodeling or even vulnerability to AF. To test this hypothesis, we isolated VLDL from healthy (normal-VLDL) and MetS individuals and intravenously injected different VLDL to mice. With this model, the *in vivo* effect of MetS-VLDL in atrial and ventricular structural remodeling and cardiac function were examined. A VLDL injection dose mimicking the normal plasma level of VLDL in human subjects was chosen (equivalent to 15 mg/dL). Cardiac parameters were measured by echocardiography after 4-week to 6-week VLDL injections. Both VLDLs caused LV mass increase (LV mass of control 92.3 ± 13.4 mg, *vs.* nVLDL 107.6 ± 16.2 mg, *vs.* msVLDL 102.7 ± 9.0 mg, *p* = 0.0553) and left ventricular dilation (LVIDd of control 3.13 ± 0.30 mm, *vs.* nVLDL 3.78 ± 0.19 mm, *vs.* msVLDL 3.79 ± 0.20 mm, *p* < 0.0001; Table 1). Decreased ejection fraction was noted in msVLDL mice (control 62.5% ± 7.75% (*n* = 10), *vs.* msVLDL 52.9% ± 9.6% (*n* = 10); *p* < 0.05 for control *vs.* msVLDL; Table 1) at week 6. Only MetS-VLDL caused significant left atrial (LA) enlargement; this change was significant after 4 weeks and progressed at 6 weeks. VLDL injection had no effect on body weight (control 23.8 ± 2.4 g (*n* = 10), *vs.* nVLDL 22.8 ± 1.7 g (*n* = 7), *vs.* msVLDL 24.6 ± 2.7 g (*n* = 10), *p* = 0.1699; Table 1 and Figure 2).

**Table 1:** Echocardiography results after six weeks of very low density lipoproteins (VLDL) injection.

Parameters	Control (*n* = 10)	nVLDL (*n* = 7)	msVLDL (*n* = 10)	*p* Value
BW (g)	23.8 ± 2.4	22.8 ± 1.7	24.6 ± 2.7	0.1699
HR (bpm)	230 ± 20	237 ± 59	259 ± 38	0.5720
**Measurement (mm)**				
Ao Root	1.71 ± 0.12	1.68 ± 0.07	1.69 ± 0.13	0.9274
LA	1.64 ± 0.23	1.84 ± 0.13	2.18 ± 0.24 ^$,#^	<0.0001
IVSd	0.91 ± 0.13	0.76 ± 0.09 *	0.78 ± 0.06 ^$^	0.0054
LVIDd	3.13 ± 0.30	3.78 ± 0.19 *	3.78 ± 0.20 ^$^	<0.0001
LVPWd	0.87 ± 0.10	0.85 ± 0.16	0.77 ± 0.11	0.2141
LVIDs	2.11 ± 0.33	2.59 ± 0.23 *	2.77 ± 0.36 ^$^	0.0003
**Calculation**				
EF (%)	62.5 ± 7.7	59.8 ± 8.1	52.9 ± 9.6 ^$^	0.0529
FS (%)	32.9 ± 5.4	31.5 ± 5.4	27.0 ± 6.5	0.0853
LV Mass (mg)	92.3 ± 13.4	107.6 ± 16.2	102.7 ± 9.0	0.0553
LVEDV (µL)	39.3 ± 8.8	61.3 ± 7.2 *	61.6 ± 7.9 ^$^	<0.0001
LVESV (µL)	15.1 ± 5.7	24.6 ± 5.4 *	29.5 ± 8.6 ^$^	0.0004

BW, body weight; HR, heart rate; Ao Root, aortic root diameter; LA, left atrium diameter; IVSd, end-diastolic interventricular septum thickness; LVIDd, end-diastolic LV internal dimension; LVPWd, end-diastolic LV posterior wall thickness; LVIDs, end-systolic LV internal dimension; EF, ejection fraction; FS, fraction shortening; LV, left ventricle; LVEDV, LV end-diastolic volume; LVESD, LV end-systolic volume; * Comparisons significant for nVLDL *vs.* Control; ^$^ Comparisons significant for msVLDL *vs.* Control; ^#^ Comparison significant for msVLDL *vs.* nVLDL.

### 2.4. Isoproterenol Challenge Induced Atrial Fibrillation (AF) in msVLDL Mice

**Figure 3:**
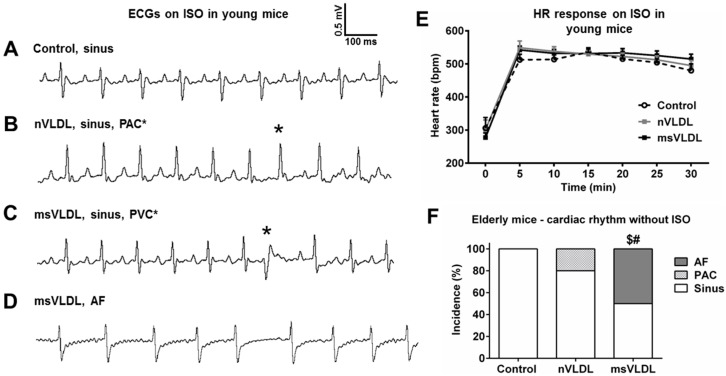
Isoproterenol-induced and unprovoked atrial fibrillation (AF) were observed only in msVLDL mice. (**A**–**D**) Representative tracings of young mice after ISO injection show abnormalities including normal regular sinus rhythm in the control group (*n* = 5), premature atrial complex (PAC) in the nVLDL group, premature ventricular complex (PVC *) and AF (absence of clear P waves and irregular RR intervals) in the msVLDL group (*n* = 5); (**E**) Heart rate responses after isoproterenol injection were not different among groups; (**F**) For elderly mice, spontaneous, unprovoked AF was noted in the msVLDL group (*n* = 6) with an incidence of 50%. PAC was observed in one mouse in the nVLDL group (*n* = 5). All control mice (*n* = 5) had sinus rhythm. $ *p* < 0.001 for msVLDL *vs.* control and # *p* < 0.001 for msVLDL *vs.* nVLDL.

Isoproterenol infusion is used for arrhythmia induction in clinical electrophysiology. For mice, we tested a single dose of intraperitoneal isoproterenol for inducing arrhythmias [19,20]. We performed electrocardiography after three, four, five and six weeks of VLDLs or saline injection for young mice. Two msVLDL mice had frequent premature atrial complexes (PACs at the 3rd and 6th week). The msVLDL mouse with PACs at week 3 died at week 4. After week 6, remaining mice were used for isoproterenol challenge. After a single injection of isoproterenol (100 ng/kg), the heart rate (HR) increased dramatically. HR responses following isoproterenol injection were similar among groups (Figure 3E). After injection of isoproterenol, one control mouse (*n* = 5), and one nVLDL mouse (*n* = 7) had PACs (Figure 3B). Two msVLDL mice had premature ventricular complexes (PVCs) and atrial fibrillation (*n* = 5) (Figure 3C,D).

### 2.5. Unprovoked Electrocardiography Showed AF in msVLDL Mice

We tested the hypothesis that spontaneous, unprovoked AF might develop if we injected msVLDL in elderly mice. Therefore, we performed the same VLDLs or saline injection experiment using 9-month-old mice. The groups, breeding and injection fashion were all the same as that used in the young mice. After 6 weeks of injection, AF was observed in 50% of msVLDL mice (*n* = 6). Sinus rhythm was noted for all nVLDL (*n* = 5) and control mice (*n* = 5) (Figure 3F). The incidence of documented AF was significantly higher in msVLDL compared to nVLDL and control mice (*p* < 0.001).

### 2.6. MetS-VLDL Caused Atrial Tissue Apoptosis

**Figure 4:**
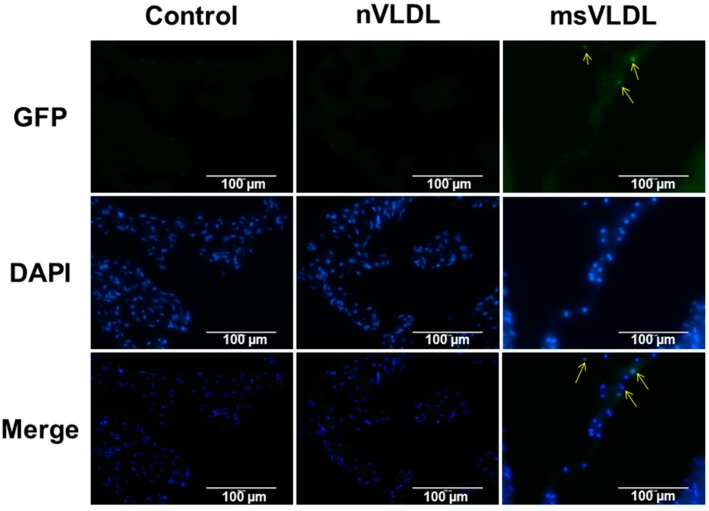
Apoptosis in atrial tissue of msVLDL mice. Representative *in situ* terminal deoxynucleotidyl transferase (TUNEL) staining of atrial tissues from control (**left**), nVLDL (**middle**), and msVLDL (**right**) (*n* = 3 for each groups). Normal nuclei with DAPI staining appear blue. Condensed or fragmented nuclei appeared bright green and indicate cells undergoing apoptosis. Arrows indicate apoptotic atrial myocytes in the msVLDL group. The scale bars indicate 100 µm.

Histological studies determined that 6 weeks’ injection of VLDL had cytotoxic effects *in vivo*. In *in situ* terminal deoxynucleotidyl transferase (TUNEL) staining, normal nuclei appear blue with DAPI staining while bright green condensed or fragmented nuclei indicate apoptosis. Apoptotic cells were observed only in msVLDL mice (*n* = 3 for each group; Figure 4).

### 2.7. Increased Lipid Accumulation in Atrial Tissue of msVLDL Mice

**Figure 5:**
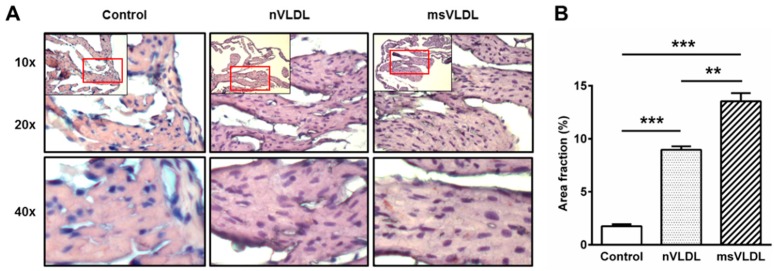
Greater lipid accumulation in atrial tissue of msVLDL mice. (**A**) Representative Oil-Red-O-stained sections of atrial tissues from control (**left**), nVLDL (**middle**), and msVLDL (**right**). Each red rectangle indicates the area to be magnified (20×). Tiny red lipid droplets in controls were few but the number increased in nVLDL and msVLDL atria. Some lipid droplets increased in size in the msVLDL; (**B**) Lipid droplets were significantly increased in the VLDL groups, especially in the msVLDL group (** *p* < 0.01, *** *p* < 0.001; *n* = 3 for each group).

To determine if normal-VLDL and MetS-VLDL were internalized into atrial tissues differently, we performed Oil-Red-O staining of atrial tissues (Figure 5). A few tiny red lipid droplets were seen in control atrial tissue. In nVLDL and msVLDL atrial tissue, lipid droplets were significantly increased (*n* = 3 for each, *p* < 0.001 *vs.* control) and lipid droplets were larger and more numerous in msVLDL atrial tissues compared to nVLDL (*p* < 0.01 for msVLDL *vs.* nVLDL), suggesting that MetS-VLDL increased lipid accumulation in atrial tissues.

## 3. Discussion

This study showed the distinctive effects of MetS-VLDL on the heart, specifically atrial myocyte apoptosis, left atrial dilation, and AF vulnerability and incidence. Physiological concentrations of MetS-VLDL caused greater lipid accumulation in atrial cells and tissue than normal-VLDL, partially via VLDL receptors.

### 3.1. Increased Lipid Accumulation and in Vivo and in Vitro Cytotoxicity of MetS-VLDL to Atrium

Greater lipid accumulation in hearts of MetS patients is associated with decreased ventricular function and cardiomyopathy [21,22]. Studies have suggested that increased triglyceride accumulation could lead to reduced energy efficiency by inducing mitochondrial damage and uncoupling, thus increasing cellular ROS, impairment of mitochondrial calcium handling, and lipoapoptosis [23]. Our study showed *in vitro* evidence that compared to normal-VLDL, MetS-VLDLs caused greater lipid uptake and cytotoxicity in parallel to increased cellular ROS in atrial cells (Figure 1 and Figure 4). Consistently, MetS-VLDL induced greater lipid accumulation and apoptosis *in vivo* (Figure 1 and Figure 5). In addition to direct cytotoxicity, we suggest that *in vivo* MetS-VLDL-induced apoptosis in atrial tissue may be also related to excessive lipid accumulation.

In normal healthy hearts, cardiac energy primarily relies on oxidation of fatty acids and to a lesser extent on glucose metabolism [24]. VLDL and chylomicrons are major sources of triglyceride for the heart. Triglyceride may be taken up through lipoprotein-lipase (LPL)-mediated lipolysis and lipoprotein receptor-mediated endocytosis. The VLDL uptake can be increased in hypoxic conditions mediated by HIF-1 α through low-density lipoprotein-related protein (LRP1) [25]. In hypoxic HL-1 cells and ischemic myocardium, VLDLR plays a major role in VLDL uptake by the heart, and closely interacts with LPL [26,27]. We found that MetS-VLDL uptake, at least partially, occurred through VLDLR (Figure 1D,E). Further experiments are needed to establish the precise contribution of LPL, VLDLR, and LRP1 in MetS-VLDL and normal-VLDL uptake in atrial and ventricular cardiomyocytes.

Normal VLDL particles are considered non-toxic to vascular cells, but apolipoprotein CIII (apoCIII)-rich VLDL exhibits atherogenicity by enhancing monocyte–endothelial cell (EC) adhesion [28,29]. ApoCIII also inhibits the uptake of VLDL by the liver. Our chemical analyses suggest that MetS-VLDL is an apoCIII- and apoE-rich lipoprotein (data not shown). Our past study showed that higher negative charged subfraction of VLDL in MetS compared to healthy subjects [17]. The negatively charged property of MetS-VLDL may be caused by post-translational modification of apolipoproteins. Further studies are mandatory to determine the biochemical changes with MetS-VLDL and the mechanism behind its negative charge.

### 3.2. MetS-VLDL Causes Cardiac Remodeling

An elegant study by Asai *et al.* [30] provided evidence that intracellular lipid moieties mediate metabolic signals leading to cardiomyocyte growth, and that marked myocardial triglyceride accumulation was associated with left ventricular dysfunction. In agreement with these findings, our study showed that ventricular dilatation with increased LV mass was caused by both normal-VLDL and MetS-VLDL injection, with worse left ventricular dysfunction after MetS-VLDL injection (Table 1). The important role of lipids in mediating left ventricular structure change has been well established, but little is known about how atrial myocytes and tissue are affected by lipid accumulation. In our animal model, the temporal change in atrial and ventricular sizes is illustrated in Figure 2. Although it is generally agreed that ventricular dysfunction produces atrial remodeling, atrial remodeling in our msVLDL mice seemed to precede the ventricular remodeling (Figure 2). The atrial remodeling in msVLDL mice was significant as early as four weeks after injection, but significant ventricular remodeling was not observed until six weeks. Therefore, we suggested that MetS-VLDL-caused atrial dilatation is not due to ventricular remodeling. Although the immunological reactions may not be avoided using human VLDLs in our mouse model, this study provides direct evidence that the *in vivo* effects of MetS-VLDL and normal-VLDL on atrial remodeling and AF vulnerability are different.

Two major remodeling mechanisms have been proposed for AF development; structural remodeling characterized by atrial fibrosis, and electrical remodeling characterized by ion channels’ modulation [31]. We performed Masson’s Trichrome staining of the young mouse atrial tissue but did not observe significant atrial fibrosis in our VLDL injection mice (data not shown). To further elucidate the cellular mechanism in MetS-VLDL-induced atrial dilation, we performed a messenger RNA microarray study of control, nVLDL, and msVLDL mice atrial tissues (data not shown). The preliminary microarray data showed that some genes involving the lipid metabolism, contractile proteins, and calcium regulation proteins were dysregulated in msVLDL atrium, suggesting that electrical remodeling may be associated with atrial dilatation in msVLDL mice. Castellano *et al.* [32] reported that in neonatal rat cardiomyocytes, high concentrations of VLDL can induce cholesteryl ester and triglyceride accumulation, and reduce sarcoplasmic reticulum Ca ATPase-2 expression, calcium transient amplitude and sarcoplasmic reticulum calcium loading. Myocardial lipid and fatty acid compositions in atrial tissues were not determined in this study. It would be interesting to study the related electrical remodeling in msVLDL mice in terms of atrial effective refractory period, calcium transient and/or electrical propagation and its relation to lipid accumulation. Although the molecular mechanisms by which MetS-VLDL leads to cardiac remodeling in parallel with lipid accumulation remain undetermined, the current study provides evidence on the scope of VLDL roles in AF pathogenesis.

### 3.3. Clinical Implications

In view of the debate on the relationship between dyslipidemia and AF in recent years, this study offers new insights addressing the discrepancy seen among diverse clinical studies. The strikingly different *in vivo* and *in vitro* effects of normal and MetS-VLDL shown in this study suggested a positive correlation for dyslipidemia and AF, especially in MetS.

The prevalence of AF increased with aging. In this study, we observed spontaneous AF in nine-month-old mice but not in young mice after receiving six-week MetS-VLDL injection. The development of cardiac fibrosis, one of the important changes in aging hearts, may lead to a better substrate for AF development [33]. Nevertheless, it is still unknown if VLDL could interact with fibrosis or any aging process of the heart, especially in the atrium.

**Figure 6:**
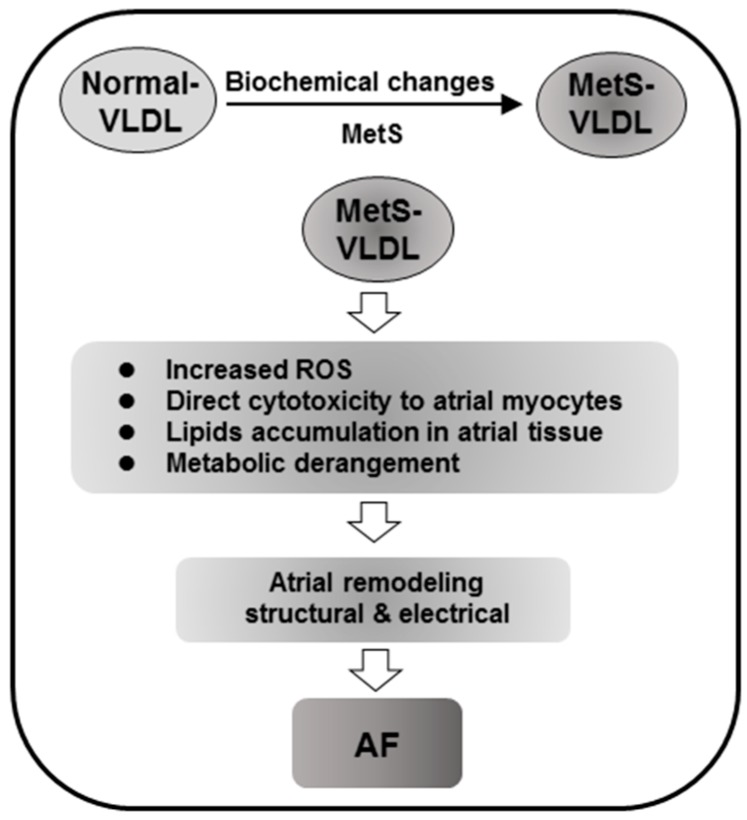
Potential mechanism by which VLDL promotes AF in MetS. In MetS, the biochemical properties of VLDL are changed. MetS-VLDL can induce cellular reactive oxygen species, atrial myocyte cytotoxicity, and excess lipid accumulation resulting in subsequent gene dysregulation corresponding to metabolic derangement. Structural and potentially electrical remodeling initiated by MetS-VLDL in concert contribute to AF vulnerability and development.

Our results show that msVLDL mice is more vulnerable to AF and suggest a pivotal role of VLDL in AF pathogenesis for MetS (Figure 6). In MetS, the biochemical properties of VLDL are changed and MetS-VLDL can induce cellular reactive oxygen species, atrial myocyte cytotoxicity, and excess lipid accumulation resulting in subsequent gene dysregulation corresponding to metabolic derangement. We suggested that both structural and electrical remodeling initiated by MetS-VLDL contribute in concert to AF. After the underlying mechanisms of the biochemical property changes of VLDL in MetS are determined, we may be able to revert bad VLDL to a normal state and thus prevent the development of AF in MetS individuals, especially in the aging population.

## 4. Material and Methods

### 4.1. VLDL Isolation

Human normal-VLDL and MetS-VLDL were isolated from pooled blood of healthy volunteers (two males and two females, age 36 ± 8) and individuals meeting criteria for MetS based on National Cholesterol Education Program–Adult Treatment Panel III guidelines (five males, age 48 ± 5) [34]. All participants gave informed consent and the study followed Helsinki Declaration principles and was approved by the Kaohsiung Medical University Hospital Ethics Review Board (the project identification code KMUH-IRB-20130351, 24 January 2014. Total VLDL (density = 0.930–1.006 g/mL) was isolated by sequential ultracentrifugation [17]. VLDL protein concentration was determined by the bicinchoninic acid method.

### 4.2. HL-1 Atrial Myocyte Culture

A murine HL-1 atrial myocyte cell line was maintained with fresh Claycomb medium in pre-coated culture flasks at 37 °C in a humidified atmosphere containing 5% CO_2_. When the cells reached confluence, splitting was performed by recommended passaging procedures. Culture medium was supplemented with 87% Claycomb medium, 2 mM/L l-glutamine, 10% fetal bovine serum, 100 U/mL penicillin, 100 µg/mL streptomycin, and 0.1 mM/L norepinephrine.

### 4.3. Cytotoxicity of VLDL to HL-1 Cells

Cell viability was evaluated by CCK-8 assay (Sigma-Adrich, St. Louis, MO, USA). HL-1 cells were treated with either normal-VLDL or MetS-VLDL in 96-well plates (5 × 10^3^ cells per well) at different test concentrations (3.125, 6.25, 12.5 and 25 mg/dL). Controls were incubated with ordinary medium. After 24 h, cells were washed with D-Hanks buffer. Two hundred microlitres of CCK-8 solution was added to each well and incubated for 3 h at 37 °C. Optical density (OD) at 450 nm was read on a Microplate Reader (Thermo, Waltham, MA, USA). Cell viability (% of control) is expressed as the percentage of (ODtest − ODblank)/(ODcontrol − ODblank), where ODtest is the optical density of the cells exposed to VLDL, ODcontrol is the optical density of the control sample and ODblank is the optical density of the wells without HL-1 cells.

### 4.4. Reactive Oxygen Species (ROS)

To measure effects of VLDLs on cytosolic oxidant activity, HL-1 cells were seeded at 8 × 10^4^ cells/well overnight and then treated with normal-VLDL or MetS-VLDL at a concentration of 25 µg/mL for 16 h (*n* = 4 for each group). The culture medium was replaced with pre-warmed D-PBS and 2′,7′-dichlorofluorescein diacetate (DCFH-DA 20 μM, Molecular Probes, Eugene, OR, USA) for 20 min of incubation. After incubation, cells were washed twice with D-PBS. A Bio-Tek FL-800 reader (BioTek, Winooski, VT, USA) was used to measure DCF fluorescence (excitation: 480 nm, emission 520 nm).

### 4.5. VLDL Uptake by HL-1 Cells

VLDLs were labeled with DiI by a modification of the method of Beisiegel *et al.* [35], incubating VLDL (1 mg/mL) in PBS—0.5% BSA with 80 µmol/L DiI in DMSO (3 mg/mL) for 8 h at 37 °C. Cells were pre-incubated in the presence or absence of anti-VLDL antibody (Novus Biologicals, Littleton, CO, USA) for 24 h and then incubated with DiI-normal-VLDL and DiI-MetS-VLDL at a final concentration of 25 μg/mL for 16 h before staining with 10 µg/mL Hoechst 33258 for fluorescent microscopic observation (*n* = 3 for each group).

### 4.6. Mice and Diet

To test *in vivo* arrhythmogenic effects of MetS-VLDL, we gave MetS-VLDL or normal-VLDL by intravenous injection in mice tail veins at a dose of 15 μg/g, three times a week for 6 consecutive weeks. The 15 μg/g dose was chosen to match the human normal VLDL concentration [34], which is between 2 and 30 mg/dL. A single 15 µg/g VLDL injection will result in a 15 mg/dL plasma level for mice. Five-week-old male C57BL/6 male mice from the National Laboratory Animal Center (Taipei, Taiwan) were maintained in a temperature-controlled facility (21–22 °C) with a 12-h light/dark cycle, free access to water and a standard chow diet. After a two-week acclimatization period, mice were randomly separated into three groups: control (*n* = 10), nVLDL (*n* = 7), and msVLDL (*n* = 11), and an equivalent 50 μL volume of phosphate buffered saline, 15 µg/g normal-VLDL or MetS-VLDL was injected respectively. Body weights were recorded weekly. To examine if AF could spontaneously occur in elderly mice, we used a batch of 9-month-old mice to perform the same VLDL injection for 6 weeks and afterwards performed electrocardiography without any provoking (control, *n* = 5; nVLDL, *n* = 5; msVLDL, *n* = 6; see below). All applicable institutional and governmental regulations concerning ethical use of animals conformed to the NIH guidelines and all animal procedures were approved by the Institutional Animal Care and Use Committee of Kaohsiung Medical University.

### 4.7. Mouse Echocardiography

*In vivo* heart function and chamber dimensions were measured after mice (control, *n* = 10; nVLDL, *n* = 7; msVLDL, *n* = 10) were anesthetized with 1.5%–2% isofluorane with a Vevo2100 (VisualSonics, Inc., Toronto, ON, Canada) small animal instrument, using a transducer with a 30 MHz frequency. Mice were fixed onto a temperature-regulated table. Left ventricular (LV) wall thicknesses (LV posterior wall (LVPW) and interventricular septum (IVS)), LV chamber dimensions (LVID) at end-diastole and end-systole (LVEDD and LVESD), and fractional shortening (FS = (LVIDd − LVIDs)/LVIDd) were determined from M-mode images. Left atrial size was determined from M-mode at end-systole. The ejection fraction (EF) and LV mass and end-diastole and end-systole volumes (LVEDV and LVESV) were calculated according to Teichholz *et al.* [36] as follows:

LVEDV=(7×LVIDd3)/(2.4+LVIDd)

LVESV=(7×LVIDs3)/(2.4+LVIDs)

EF(%)=(LVEDV−LVESV)/LVEDV

$$\mathrm{LV Mass}=1.05\times \left[{\left(\mathrm{LVIDd}+\mathrm{LVPWd}+\mathrm{IVSd}\right)}^{3}-{\left(\mathrm{LVIDd}\right)}^{3}\right]$$

### 4.8. Isoproterenol-Challenged Electrocardiography for Young Mice

After 6 weeks of VLDL or saline injection, a single dose of 100 ng/kg isoproterenol hydrochloride (Sigma-Aldrich, St. Louis, MO, USA) was injected intraperitoneally in young mice (control, *n* = 5; nVLDL, *n* = 7; msVLDL, *n* = 5). After 5 min, an electrocardiogram (ECG) was recorded with the same procedure as for elderly mice (see below).

### 4.9. Unprovoked Electrocardiography for Elderly Mice

After 6 weeks of VLDL or saline injection, platinum electrodes were inserted subcutaneously in the limbs and connected to a custom-built ECG amplifier under anesthesia with intraperitoneal injection of pentobarbital 0.5–1.0 µg/g. The recording was initiated after the tracing was stable and lasted for 5 min for each test.

### 4.10. Oil Red O (ORO) Staining of Atrial Tissue and Quantification

Lipid accumulation was assessed by ORO staining of 10 μm paraffin sections of atrial tissues fixed in phosphate-buffered 4% paraformaldehyde. Color images were acquired using a micropublisher 3.3 RTV camera, saved as TIFF files, and analyzed using Image J software. A 100-µm^2^ grid overlay was generated over each image, and the area fraction (%), defined as (points over ORO)/(points over image)/100, was determined. The values were derived from the average over the entire area of atrial tissue in multiple animals for each experimental group (*n* = 3 for each).

### 4.11. In Situ Terminal Deoxynucleotidyl Transferase (TUNEL) Assay of Atrial Tissues

Ten μm paraffin sections of atrial tissues were fixed in phosphate-buffered 4% paraformaldehyde, incubated with proteinase K (20 μg/mL) for 30 min, washed with PBS and covered with 50 μL of terminal deoxynucleotidyl transferase reaction mixture containing 5 units of terminal deoxynucleotidyl transferase, 1.5 mM CoCl_2_, and 0.5 mM 2′-deoxyuridine-5′-triphosphate coupled to biotin (biotin-16-dUTP). The sections were exposed to solution containing 5 μg/mL of FITC-labeled Extravidin (Sigma-Adrich, St. Louis, MO, USA), 4× concentrated SSC buffer, and 5% nonfat dry milk for 30 min, washed with PBS, and finally with 4′,6′-diamidino-2-phenylindole (DAPI) dye to visualize nuclei. Staining was performed in quadruplicate for each group (*n* = 3 for each). Double-stranded DNA cleavage was determined by green (FITC) nuclear fluorescence (Sigma-Adrich, St. Louis, MO, USA).

### 4.12. Data Analysis and Statistics

Data were expressed as means ± SD; n indicates the number of cell samples or mice. One-way ANOVA and Tukey’s multiple comparisons test were used to compare values among groups. Chi-square test was used to determine the difference of AF incidence among groups. Statistical significance was considered as *p* value ≤0.05.

## 5. Conclusions

In this study, we showed for the first time that physiological concentrations of MetS-VLDL cause atrial myocyte cytotoxicity, excess lipid accumulation and apoptosis in atria, resulting in left atrial enlargement, and that these changes are associated with increased incidence of AF. These findings have potential clinical impact. Our results suggest that VLDL may serve as a potential mediator of MetS-related AF and thus be promising a therapeutic target for AF prevention in MetS.

## Abbreviations

The following abbreviations are used in this manuscript

MetS: metabolic syndrome
AF: atrial fibrillation
VLDL: very-low-density lipoproteins
nVLDL: normal-VLDL treated group
msVLDL: MetS-VLDL treated group
ORD: oil red O
ISO: isoproterenol
